# Crystal Structure of the Chloroplastic Glutamine Phosphoribosylpyrophosphate Amidotransferase GPRAT2 From *Arabidopsis thaliana*

**DOI:** 10.3389/fpls.2020.00157

**Published:** 2020-02-27

**Authors:** Xueli Cao, Bowen Du, Fengjiao Han, Yu Zhou, Junhui Ren, Wenhe Wang, Zeliang Chen, Yi Zhang

**Affiliations:** ^1^Beijing Advanced Innovation Center for Soft Matter Science and Engineering, Beijing Key Laboratory of Bioprocess, Beijing University of Chemical Technology, Beijing, China; ^2^Department of Computational Chemistry, National Institute of Biological Sciences, Beijing, China; ^3^Key Laboratory of Livestock Infectious Diseases in Northeast China, Ministry of Education, College of Animal Science and Veterinary Medicine, Shenyang Agricultural University, Shenyang, China

**Keywords:** chloroplastic glutamine phosphoribosylpyrophosphate amidotransferase, herbicide, X-ray crystallography, competitive inhibition, Arabidopsis thaliana, DAS734

## Abstract

Chloroplastic glutamine phosphoribosylpyrophosphate amidotransferase (GPRATase) catalyzes the first committed step of *de novo* purine biosynthesis in *Arabidopsis thaliana*, and DAS734 is a direct and specific inhibitor of AtGPRAT, with phytotoxic effects similar to the leaf beaching phenotypes of known AtGPRAT genetic mutants, especially *cia1* and *atd2*. However, the structure of AtGPRAT and the inhibition mode of DAS734 still remain poorly understood. In this study, we solved the structure of AtGPRAT2, which revealed structural differences between AtGPRAT2 and bacterial enzymes. Kinetics assay demonstrated that DAS734 behaves as a competitive inhibitor for the substrate phosphoribosyl pyrophosphate (PRPP) of AtGPRAT2. Docking studies showed that DAS734 forms electrostatic interactions with R264 and hydrophobic interactions with several residues, which was verified by binding assays. Collectively, our study provides important insights into the inhibition mechanism of DAS734 to AtGPRAT2 and sheds light on future studies into further development of more potent herbicides targeting *Arabidopsis* GPRATases.

## Introduction

*De novo* purine nucleotide biosynthesis is important for plant growth and development. This essential pathway in plant metabolism plays series of key roles, including providing purine precursors for DNA and RNA, B-class vitamins, and plant hormones (Senecoff et al., 1996; Herz et al., 2000; Moffatt and Ashihara, 2002). In addition, several vital coenzymes, such as NAD, FAD, and FMN, are derived from this pathway and phosphoribosylpyrophosphate (PRPP) is utilized in the biosynthesis of all these coenzymes. (Hanson and Gregory, 2002; Boldt and Zrenner, 2003; Zrenner et al., 2006; Hove-Jensen et al., 2017). As the key regulatory enzyme in the pathway, researches into the glutamine phosphoribosylpyrophosphate amidotransferase (GPRATase) of *Arabidopsis thaliana* have shown the important role of *de novo* purine biosynthesis in chloroplast development or function as well as cell division (Hung et al., 2004; Yang et al., 2015).

GPRATase catalyzes the first committed step of purine biosynthesis (Smith et al., 1994; Smith and Atkins, 2002), transforming phosphoribosylpyrophosphate (PRPP) to phosphoribosylamine (PRA) with amide group of glutamine as nitrogen source. The entire catalytic reaction is shown in **Figure S1** (Walsh et al., 2007). Ten enzymatic reactions are required in the purine biosynthesis pathway to generate inosine monophosphate (IMP). Out of these ten enzymatic transformations, six enzymes including GPRATase are common, and four steps could be catalyzed by different enzymes in various organisms (Zhang et al., 2008). Two aminotransferases are involved in this pathway, GPRATase with an N-terminal nucleophile-glutaminase and PurLQS with a triad glutaminase activity, respectively (Zhang et al., 2008). GPRATase is also subjected to feed-back inhibition by purine nucleotides and thus forms the important control over *de novo* purine biosynthesis (Walsh et al., 2007). Genes encoding GPRATase have been found in bacteria, Eukarya, and Archaea. However, only the enzymes from *Escherichia coli* (*E. coli*) and *Bacillus subtilis* (*B. subtilis*) have been characterized through structural and biochemical studies (Smith, 1998). The two enzymes are both homo-tetramers and are representative of two classes of GPRATases. The *B. subtilis* GPRATase (BsGPRAT) is synthesized with an N-terminal propeptide and an Fe-S center, whereas the *E. coli* GPRATase (EcGPRAT) has neither of them. Three homologs of GPRATases (AtGPRAT1-3) exist in the Arabidopsis genome with differentially expression pattern in various plant tissues (Ito et al., 1994; Boldt and Zrenner, 2003; Hung et al., 2004; van der Graaff et al., 2004; Woo et al., 2011), which is quite different from most of the other enzymes in purine biosynthesis present with a single isoform (Boldt and Zrenner, 2003). Previous biochemical and genetic studies have confirmed that AtGPRAT2 (At4g34740) is the major isoform expressing in leaves (Hung et al., 2004).

AtGPRAT2 was localized in the stroma of chloroplasts in *Arabidopsis* leaf cells (Hung et al., 2004), and recently was further confirmed in the nucleoid of chloroplasts (Yang et al., 2015). The AtGPRAT2-deficient mutants *cia1* (chloroplast import apparatus1), *dov1* (differential development of vascular associated cells 1), *dg169* (delayed greening 169), *alx13* (altered APX2 expression 13), and knock out mutant *atd2* (amidotransferase-deficient2) showed growth retardation and bleached seedling phenotype which was also regarded as leaf chlorosis, but could survive under low light condition (Hung et al., 2004; van der Graaff et al., 2004; Woo et al., 2011; Rosar et al., 2012; Yang et al., 2015). The bleached new leaves imply damage by photooxidative effects or harmful effects on chloroplast biogenesis. The phenotype could be restored to wild-type by the addition of AMP or IMP, but not cytokinin or nicotinamide adenine dinucleotide (NADH), to the medium (Hung et al., 2004; van der Graaff et al., 2004). The leave number of *cia1* mutant is only half of wild-type plants, while with slightly smaller cell size. In addition, the protein-import efficiency of the chloroplasts isolated from *cia1* mutant is only less than 50% compared with wild-type chloroplasts, but the import efficiency cannot be rescued by adding ATP and GTP. These phenotypes suggested that *de novo* purine biosynthesis is also vital for cell division and chloroplast biogenesis. A recent research into *dg169* mutant indicated AtGPRAT2 featured in early chloroplast development through maintaining PEP (plastid-encoded RNA polymerases) function, thus sustaining normal transcription and translation (Yang et al., 2015).

Currently, very few small molecules have been known to act directly and specifically on the important purine biosynthetic pathway, especially in the initial reactions of the pathway, while the novel phenyltriazole acetic acid [5-(4-chlorophenyl)-1-isopropyl-1H-[1,2,4]triazol-3-yl]-acetic acid (DAS734) compound is an exception in contrast to nonspecific inhibitors such as azaserine, acivicin, and 6-diazo-5-oxo-L-norleucine (Lyons et al., 1990). DAS734 shows herbicidal activity on the seedlings of a variety of dicotyledonous weeds, producing bleaching of newly emerged leaves and root inhibition, phenocopying *AtGPRAT2* mutants with variegated bleached-white appearance (Hung et al., 2004; van der Graaff et al., 2004). However, the phenotype it induced is different from many other herbicidal inhibitors of primary metabolism. It is lethal when *Arabidopsis* seedlings were treated with more than 5 μM DAS734. In particular, DAS734 is effective on Arabidopsis, but with no inhibitory activity on *E. coli*, cyanobacteria, green algae, or yeasts. The phytotoxic effects of DAS734 can be alleviated only by the end product adenine and its derivatives (Walsh et al., 2007), similar to the phenotypes of GPRATase mutants. The combination of genetic and biochemical study has confirmed that the phytotoxicity of DAS734 results from direct inhibition of AtGPRATases. Therefore, treatment by DAS734 is equal to knockout mutants lacking *AtGPRAT2* and *AtGPRAT3* or even all GPRAT activity, thus overcoming GPRAT genetic redundancy. Therefore, DAS734 has been established as a specific biochemical probe for plant purine biosynthesis and especially useful in analyzing how the disrupted GPRATases impose influences on impaired chloroplast biogenesis and new leaf bleaching. In addition, DAS734 could also be used as a novel bleaching herbicide (Walsh et al., 2007). Despite the importance of plant GPRATases and its inhibitor DAS734, studies into the structure of plant GPRATases and inhibition mechanism of DAS734 have been enigmatic.

Here, we report the crystal structure of AtGPRAT2 and investigate the binding mode of DAS734 through molecular biochemical and docking studies. Our results indicated that AtGPRAT2 folds more like BsGPRAT than EcGPRAT. Compared with bacterial enzymes, AtGPRAT2 also exhibits different features in the conformations of active site loops. Molecular docking and kinetics studies suggested that DAS734 inhibits AtGPRAT2 through a competitive manner with respect to PRPP. Together, our study offers insights into the inhibition mechanism of DAS734 on AtGPRAT2 and will facilitate further development of more potent herbicides targeting *Arabidopsis* GPRATases.

## Materials and Methods

### Materials

The pfu polymerase, the two restriction enzymes Nde I and Xho I and T4 DNA ligase were all purchased from Thermo Fisher. Ni-NTA beads were purchased from QIAGEN and the Superdex-200 column was purchased from GE Healthcare. DAS734 was synthesized as described in Walsh et al. (2007).

### Cloning, Expression, and Purification

The gene of AtGPRAT2 was amplified from the complementary DNA (cDNA) of *Arabidopsis thaliana* and sub-cloned into the bacterial expression vector pET22b, to produce a C-terminal His-tagged fusion protein. The AtGPRAT2 mutants were generated by two-step PCR and were subcloned, overexpressed and purified in the same way as wild-type protein. The protein was expressed in *E. coli* strain BL21 and induced by 0.2 mM isopropyl-β-D-thiogalactopyranoside (IPTG) when the cell density reached an OD_600nm_ of 1.0. After growth at 16°C for 18 h, the cells were harvested, re-suspended in lysis buffer (50 mM Tris pH 8.0, 10 mM imidazole, and 300 mM NaCl) and lysed by sonication. Recombinant His-tagged protein was purified by Ni-affinity column chromatography and was further subjected to gel filtration chromatography (Superdex-200 column) in buffer containing 10 mM Tris-HCl pH 8.0, 200 mM NaCl, 5 mM dithiothreitol (DTT). The puriﬁed protein was analyzed by sodium dodecyl sulfate polyacrylamide gel electrophoresis (SDS–PAGE). The fractions containing the target protein were pooled and concentrated to 20 mg/ml.

### Crystallization, Data Collection, Processing, and Structure Determination

Crystallization screening was performed by the sitting-drop vapor-diffusion method at 291 K. 1 μl protein solution (20 mg/ml) was mixed with an equal volume of reservoir solution in 48-well plates and the drops were equilibrated against 80 μl reservoir solution. Crystals appeared from several conditions, out of which HR2-110 No. 11 from Hampton Research was further taken to do crystal optimization. Crystals of the best diffraction quality appeared in about 1 week, which were used for data collection. The final optimized condition was 0.1 M Sodium citrate tribasic pH 5.6, 1.5 M ammonium phosphate monobasic, and 0.1 M citric acid pH 3.4.

All the data were collected at SSRF beamline BL17U1 and BL19U1, integrated and scaled using the HKL2000 package (Otwinowski, 1997). The initial model was solved by molecular replacement by the PHASER program from the CCP4 suite (Collaborative Computational Project, 1994) and refined manually using COOT (Emsley and Cowtan, 2004). The structure was further refined with PHENIX (Adams et al., 2002) using non-crystallographic symmetry and stereochemistry information as restraints. The final structure was obtained through several rounds of refinement.

### Docking Studies

The inhibitor DAS734 was docked onto AtGPRAT2 using UCSF DOCK 3.7 (Coleman et al., 2013). The binding site was defined as the set of protein residues which have at least one heavy atom within 10 Å of the residue R264. A flexible-receptor docking protocol was applied to treat binding-site side-chain flexibility (Li et al., 2019). Multiple poses were generated for structural filtering and conformational clustering (Peng et al., 2013). All the survived poses were then submitted for MM-GB/SA refining and rescoring with OPLS all-atom force field (Banks et al., 2005) using UCSF PLOP (Jacobson et al., 2004). The conformation of 472-477 loop was rebuilt and minimized along with the ligand considering its ambiguous electron density.

### AtGPRAT2 Enzymatic Activity Assay

Purified wild-type and mutant AtGPRAT2s were desalted to a buffer containing 10 mM Tris pH 7.8, 200 mM NaCl, 5 mM MgCl_2_, and 10 mM DTT. AtGPRAT2 activity was assayed by measuring the production of Glu from Gln. Glu production was determined by coupling the glutamate dehydrogenase (GDH) reaction (Messenger and Zalkin, 1979), in which Glu was oxidized and NAD^+^ was simultaneously reduced to NADH. Then, we continuously monitored NADH produced at 340 nm every 1 second, using a UV-VIS SPECTROPHOTOMETER UV-2450 (SHIMADZU). The standard assay for enzymatic activity measurement contained 37.5 mM NAD^+^, 10 mM Gln, 2.5 mM PRPP, 247.5 units/ml GDH, 110 mM potassium phosphate buffer pH 8.0, and about 0.125 mg/ml AtGPRAT2 in a total volume of 300 µl. The reaction was initiated by the addition of AtGPRAT2 and the enzymatic activities were determined utilizing extinction coefficient for NADH of 6,220 cm^−1^ M^−1^ at 340 nm. For calculating the kinetic constants *K*_m_ and *V*_max_, we held the Gln concentration constant at 10 mM, and fitted the data to the appropriate equations using GraphPad Prism software with mixed inhibition model. Effects of inhibition by DAS734 were determined by the standard assay in which DAS734 at concentration of 0, 10, 20, and 40 µM was added respectively. Then we calculated the *K*i using the GraphPad Prism software with mixed inhibition model.

### Isothermal Titration Calorimetry Binding Assay

The dissociation constants of binding reactions of wild-type and mutants of AtGPRAT2 with DAS734 were determined by isothermal titration calorimetry (ITC) using a MicroCal ITC200 calorimeter. Proteins were desalted into the working buffer [20 mM 4-(2-hydroxyethyl)-1-piperazineethanesulfonic acid (HEPES) pH 7.5, 200 mM NaCl]. The titration was carried out with 19 successive injections of 2 μl DAS734 at the 0.3 mM concentration, spaced 125 s apart, into the sample cell containing AtGPRAT2 at the 0.04 mM concentration at 25°C. The Origin software was used for baseline correction, integration, and curve ﬁtting to a single site binding model.

## Results

### Overall Structure of AtGPRAT2

**Figure 1:**
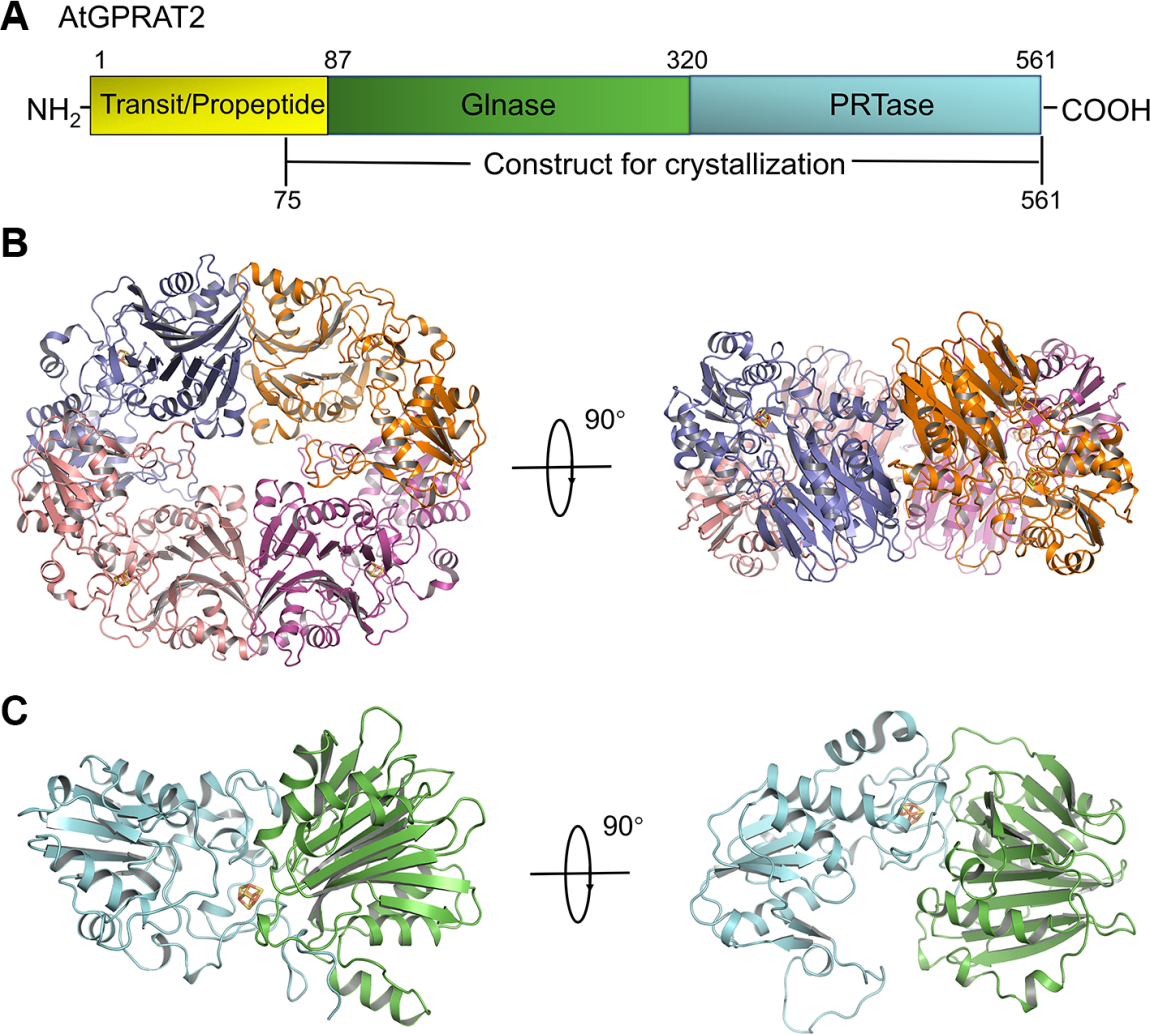
Overview of AtGPRAT2. **(A)** The domain architecture of AtGPRAT2, and the construct for crystallization is indicated in the bottom. **(B)** Cartoon model of the tetramer conformation of AtGPRAT2. The protomers are shown in different colors and the 4Fe-4S cofactor is shown in sticks. Two views are shown. **(C)** Cartoon model of one protomer of AtGPRAT2. The Glnase domain and PRTase domain are colored in green and cyan, respectively. Two views are shown.

**Figure 2:**
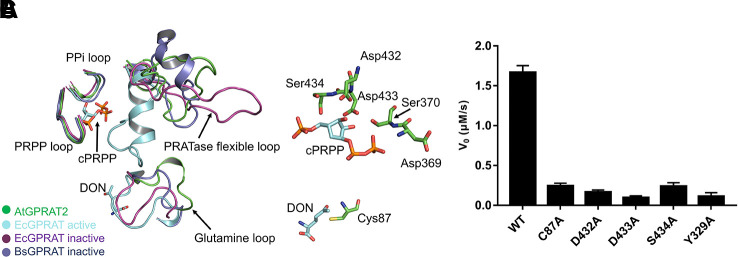
Active site of AtGPRAT2. **(A)** Structural superimposition of AtGPRAT2, EcGPRAT in the active [Protein Data Bank (PDB): 1ECC] and inactive (PDB: 1ECF) conformation, and *Bacillus subtilis* glutamine phosphoribosylpyrophosphate amidotransferase (BsGPRAT) in the inactive conformation (PDB: 1GPH). Only the indicated loop regions are shown in cartoon models. The ligands in 1ECC are shown as sticks. cPRPP, carbocyclic phosphoribosyl pyrophosphate (PRPP). DON, 6-diazo-5-oxo-L-*nor*-leucine, is an analog of substrate glutamine. The conserved loop regions are marked. **(B)** An enlarged view of the active site of AtGPRAT2. Several active site residues (green) and the substrate analogs cPRPP and DON in the aligned structure of EcGPRAT in the active conformation (PDB: 1ECC) are shown in sticks. **(C)** Activity assay with the wildtype and several active site mutants of AtGPRAT2. Error bars are standard error of the mean (SEM) values of three independent experiments.

We solved the crystal structure of a recombinant AtGPRAT2^75-561^ without the predicted chloroplast transit peptide at 3.07 Å resolution (**Figure 1A** and **Table S1**). This recombinant protein exhibited high GPRATase activity which was measured by the enzymatic assay (**Figure 2C** and **Table S2**). The crystal belonged to the P3121 space group and two AtGPRAT2 molecules were found in the asymmetric unit, each with a 4Fe-4S cofactor. However, the PISA (Proteins, Interfaces, Structures and Assemblies) server (Krissinel and Henrick, 2007) indicated that AtGPRAT2 exists as a homo-tetramer in crystal as other members of this family (**Figure 1B**). It was known that all eukaryotic and many microbial GPRATs harbor a short N-terminal propeptide, which is autocatalytically cleaved to yield a conserved N-terminal Cys. Walsh et al. also showed that in recombinant AtGPRAT2 expressed in *E. coli*, the propeptide was removed to expose the N-terminal catalytic Cys87 (Walsh et al., 2007). Consistent with this, in the AtGPRAT2 structure, the first residue with interpretable electron density was the predicted N-terminal Cys87, the mutation of which abolished the activity of the enzyme (**Figure 2C**). Moreover, the visible electron density corresponded to the AtGPRAT2 fragment spanning residues from 87 to 546. Similar to members of this family, the overall structure of AtGPRAT2 folds into an N-terminal glutaminase (Glnase) domain (residues 87-320) and a C-terminal phosphoribosyltransferase (PRTase) domain (residues 321-546) (**Figures 1A, C**). Structural comparison indicated that AtGPRAT2 adopts an inactive, open conformation as compared to the structures of the EcGPRAT in the presence or absence of the PRPP analog carboxylic PRPP (cPRPP), which represent the active and inactive state, respectively (**Figure S2**).

### Structural Comparison With Other GPRATases

The reaction catalyzed by GPRATases is carried out as two half-reactions by two separate domains, in tight allosteric communication between each other (Smith, 1998; Bera et al., 2000). One is the Glnase domain where Gln is hydrolyzed to yield ammonia. And then, the ammonia is transferred through a ~20-Å hydrophobic channel to a distal PRTase domain, in which PRA is synthesized from PRPP and ammonia. Formation of the ammonia channel is a characteristic of the glutamine amidotransferases, which catalyze the synthesis of different aminated products (Mouilleron and Golinelli-Pimpaneau, 2007). Unlike the carbamoyl-phosphate synthetase (CPS) and asparagine synthetase B (AsnB), in which the channel forms in the absence of an acceptor, the formation of the channel in AtGPRAT2 requires the binding of acceptor and closing of the PRTase flexible loop, similar as EcGPRAT and BsGPRAT (**Figure S2**). Whereas the exact shape of the channel still awaits the structure of AtGPRAT2 complexed with PRPP or cPRPP, one can get information from that of EcGPRAT based on the high identity of the channel-lining residues between the two proteins (Bera et al., 2000) (**Figure S3**). Then we compared the structures of the essential catalytic motifs of the two domains between AtGPRAT2 and bacterial enzymes (**Figure 2A**). In the Glnase domain, a conserved glutamine loop binds glutamine during the first half reaction. In contrary to the glutamine loop of EcGPRAT, which is in a closed conformation no matter glutamine binds or not, the glutamine loop of AtGPRAT2 exhibits an open conformation more similar to that of BsGPRAT (**Figure 2A**). Nevertheless, the glutamine loop of AtGPRAT2 opens to an extent larger than that of BsGPRAT, and forms a 3_10_ helix (**Figure 2A**). In the PRTase domain, three loops in core fold are important for PRPP binding and catalysis (**Figure S3**). The “PRPP loop” binds the ribose-5-phosphate group of PRPP and the adjacent “PPi loop” interacts with the pyrophosphate of PRPP. The PRTase flexible loop undergoes remarkable conformational change during the catalysis, which closes over the active site as a helix when PRPP binds and is generally open when the substrate binding site is free. Structural superimposition indicated that both PRPP and PPi loops exhibit highly conserved folds, and single mutations of three residues Asp432, Asp433 and S434 within the PRPP loop all markedly decreased the activities of AtGPRAT2 (**Figure 2C**). The PRTase flexible loops of AtGPRAT and bacterial enzymes display different open conformations, consistent with the disordered feature of this loop. This is also confirmed by the high B-factors of the PRTase flexible loop region of AtGPRAT2 (**Figure S4A**). The C-terminal helix (residues 471-492 in EcGPRAT) is also an important feature of EcGPRAT (**Figure S2**) (Smith, 1998). However, similar as BsGPRAT, AtGPRAT2 does not contain the helix and the corresponding region folds as a flexible loop (**Figures S2** and **S4**).

### DAS734 Inhibits AtGPRAT2 in a Competitive Manner With Respect to Phosphoribosyl Pyrophosphate

**Figure 3:**
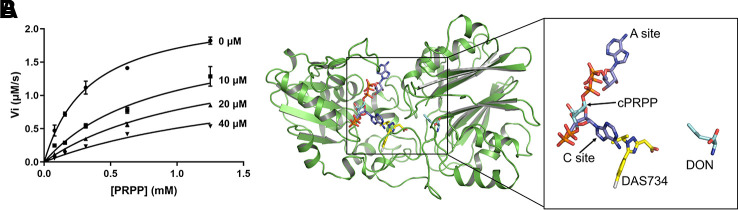
The inhibition mode of DAS734 on AtGPRAT2. **(A)** Inhibition kinetics showing that DAS734 is a competitive AtGPRAT2 inhibitor with respect to phosphoribosyl pyrophosphate (PRPP). The labels indicate different DAS734 concentrations. The data was fitted to the mixed inhibition model in GraphPad Prism software, and the alpha value of 12.56 calculated by this model indicated a competitive inhibition. Error bars are standard error of the mean (SEM) values of three independent experiments. **(B)** Structural superimposition of AtGPRAT2 with docked DAS734, EcGPRAT in the active [Protein Data Bank (PDB): 1ECC] and BsGPRAT in the inactive conformation (PDB: 1GPH). The cPRPP and 6-diazo-5-oxo-L-*nor*-leucine (DON) in the structure of 1ECC are shown as cyan sticks. The two AMP molecules in the A and C sites are shown as slate sticks. The docked DAS734 is shown in yellow sticks with the chlorine atom colored in gray.

Studies into GPRATases from bacteria and eukaryotes found that the enzyme undergoes feedback inhibition by the end products of the purine biosynthetic pathway, such as AMP, GMP, ADP, and GDP (Chen et al., 1997). Recently, the growth regulator, guanosine tetraphosphate (ppGpp) was also shown to inhibit EcGPRAT in a competitive manner (Wang et al., 2019). DAS734 has been shown to be a slow, tight-binding inhibitor for both AtGPRAT2 and AtGPRAT3 (Walsh et al., 2007). To characterize the inhibition mode of DAS734, we performed *in vitro* enzymatic kinetics assay of AtGPRAT2. In the study of Walsh et al. they determined the *K*m for glutamine as 1.34 mM. Here in this study, we set up a coupled-enzyme reaction with AtGPRAT2 and glutamate dehydrogenase to monitor the production of glutamate by AtGPRAT2 continuously. Using this system, we determined the *K*_m_ of AtGPRAT2 for PRPP as 0.35 mM (**Figure S5**). And then, the kinetics of AtGPRAT2 was tested under different concentrations of DAS734. The inhibition kinetics (**Figure 3A** and **Table S3**) showed that DAS734 behaves like a competitive inhibitor with respect to PRPP with a *K*i of 5.293 µM.

### The Binding Site of DAS734

Two inhibitor binding sites are located in the PRTase domain, an A (allosteric) site and a C (catalytic) site (**Figure 3B**) (Chen et al., 1997). The A site overlaps the site for the pyrophosphate of PRPP, and the C site overlaps the site for the ribose-5-phosphate part of PRPP. Synergistic inhibition of GPRATases was also observed by a combination of adenine and guanine nucleotides (Chen et al., 1997; Smith, 1998). However, ppGpp was found to bind at the interface between the Glnase domains of two protomers within the tetramer in a ratio of 1:2 (ppGpp to EcGPRAT), a position totally different from the known A and C sites (Wang et al., 2019).

To get insights into the binding site of DAS734 within AtGPRAT2, we attempted to get the complex structure of AtGPRAT2 with DAS734 but still failed. Therefore, we turned to docking assays to analyze the binding sites of DAS734. Previous studies have shown that the R264K mutation of AtGPRAT2 will render the enzyme highly insensitive to DAS734 (Walsh et al., 2007). Therefore, the docking region was set around R264 of AtGPRAT2. The results showed that the binding site of DAS734 partially overlaps with the known C site in the PRTase domain (**Figure 3B**).

**Figure 4:**
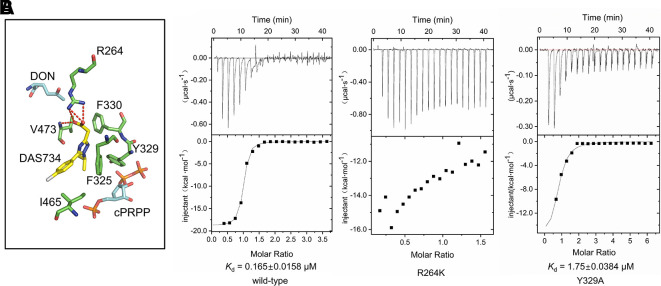
The binding site of DAS734. **(A)** Structural superimposition of AtGPRAT2 with docked DAS734 and EcGPRAT in the active [Protein Data Bank (PDB): 1ECC] conformation. The AtGPRAT2 residues involved in DAS734 binding are shown as sticks. The carbocyclic phosphoribosyl pyrophosphate (cPRPP) and 6-diazo-5-oxo-L-*nor*-leucine (DON) molecules in EcGPRAT are colored as in **Figure 3B**. Electrostatic interactions and hydrogen bonds are shown as red dashed lines. **(B)** Interaction of AtGPRAT2 mutants and DAS734 as detected by isothermal titration calorimetry (ITC). The ITC curves are shown. The calculated *K*_d_ mean ± SE values are indicated. The top plots represent time, and the bottom plots represent molar ratio. These experiments were performed twice with equivalent results.

The binding of DAS734 to AtGPRAT2 was found to involve both hydrophilic and hydrophobic interactions (**Figure 4A**). The guanidyl group of R264 forms electrostatic interactions with the carboxyl group of the acetate moiety. Moreover, the oxygen atom of the carboxyl group also forms a hydrogen bond with the amide nitrogen atom of V473. In addition, the sidechains of F325, Y329, F330, and I465 form hydrophobic interactions with DAS734 (**Figure 4A**). To validate the docking results, we performed the isothermal titration calorimetry (ITC) assay with wildtype, R264K and Y329A mutants of AtGPRAT2. The results showed that the R264K mutation abolished the binding of DAS734 to AtGPRAT2, but the Y329A mutation only slightly decreased DAS734 binding (**Figure 4B**). This suggested that the electrostatic interactions from R264 contribute much to the binding of DAS734, while the hydrophobic interaction from Y329 may play a minor role. In the previous study, P476S, P265S, and G371S mutations could also confer resistance to DAS734 at levels of around 60, 6, and 5-fold, respectively. We also analyzed these three sites in the structure of AtGPRAT2 docked with DAS734 (**Figure S6**). P476 is adjacent to the binding pocket of DAS734 and on the same loop with V473, whose amide nitrogen atom forms a hydrogen bond with the carboxyl group of the acetate moiety of DAS734. P265S mutation may affect the location of R264, thus interfering with DAS734 binding. G371 is in the PRTase domain and away from the binding pocket of DAS734. The G371S mutation may confer resistance to DAS734 through allosteric communications between the two domains.

## Discussion

GPRATase catalyzes the first committed step of *de novo* purine biosynthesis. Although genes encoding GPRATase have been found in bacteria, Eukarya, and Archaea, only the GPRATase from *E. coli* and *B. subtilis* have been characterized through structural and biochemical studies. AtGPRAT2 is the primary isoform among the three homologs of GPRATases (AtGPRAT1-3) in *Arabidopsis thaliana*. To our knowledge, the 3.07 Å crystal structure of AtGPRAT2 in this study offered the first description of the structure of GPRATases in plants, with several structural differences, especially in the catalytic motifs, when compared with bacterial enzymes.

Combined with GPRATase kinetic assay, ITC and molecular docking, we showed that DAS734 behaved as a competitive inhibitor for PRPP and proposed the binding site of DAS734. Notably, the binding site of DAS734 does not interfere with the interface between each monomer in the tetrameric structure (**Figure S7**). Interestingly, the docking site of DAS734 does not directly overlaps the binding site of PRPP (**Figure 3B**), raising the question of how DAS734 behaves its competitive function with respect to PRPP. We proposed that the binding of DAS734 might induce conformational changes surrounding it, thus influencing the binding of PRPP. Supporting this notion, Y329 is a highly conserved residue among GPRATases and its counterpart in EcGPRAT, Y258, interacts with the pyrophosphate group of PRPP (Krahn et al., 1997). Consistently, the Y329A mutation almost abolished the activity of AtGPRAT2 (**Figure 2C**). In contrast, the EcGPRAT counterparts of the interacting residues of DAS734 do not contribute to glutamine binding (data not shown). Walsh et al. also showed that DAS734 is a noncompetitive inhibitor with respect to glutamine (Walsh et al., 2007). Therefore, the binding of DAS734 to AtGPRAT2 might cause movements of its surrounding residues, especially Y329, thus preventing PRPP binding. However, this still needs to be tested in the future studies.

DAS734 could be used not only as a specific biochemical probe to help analyze how disruption of GPRATases damage the chloroplast development or function and lead to the leaf bleaching, but also as a novel bleaching herbicide(Walsh et al., 2007). In the future, more efforts should be made in the development of more potent herbicides targeting *Arabidopsis* GPRATases according to the inhibition mechanism of DAS734 to AtGPRAT2.

## Data Availability Statement

The coordinate and structure factors for AtGPRAT2 have been deposited in the Protein Data Bank (PDB) under the accession code: 6LBP. All other data are available from the corresponding author upon reasonable request.

## Author Contributions

YiZ conceived, designed and supervised the project. XC, BD, FH, JR, WW and ZC purified the proteins, grew and optimized the crystals, collected the diffraction data, solved the structure and performed enzymatic analysis. YuZ performed docking studies. YiZ analyzed the data and wrote the paper with the help of all the authors.

## Funding

This work was supported by the National Natural Science Foundation of China (31822012), the Fundamental Research Funds for the Central Universities (XK1802-8 and ZY1934) and the Beijing Natural Science Foundation (5204038) to YiZ.

## Conflict of Interest

The authors declare that the research was conducted in the absence of any commercial or financial relationships that could be construed as a potential conflict of interest.
