# Corrigendum: Crystal Structure of the Chloroplastic Glutamine Phosphoribosylpyrophosphate Amidotransferase GPRAT2 From *Arabidopsis thaliana*

**DOI:** 10.3389/fpls.2021.826504

**Published:** 2021-12-23

**Authors:** Xueli Cao, Bowen Du, Fengjiao Han, Yu Zhou, Junhui Ren, Wenhe Wang, Zeliang Chen, Yi Zhang

**Affiliations:** ^1^Beijing Advanced Innovation Center for Soft Matter Science and Engineering, Beijing Key Laboratory of Bioprocess, Beijing University of Chemical Technology, Beijing, China; ^2^Department of Computational Chemistry, National Institute of Biological Sciences, Beijing, China; ^3^Key Laboratory of Livestock Infectious Diseases in Northeast China, Ministry of Education, College of Animal Science and Veterinary Medicine, Shenyang Agricultural University, Shenyang, China

**Keywords:** chloroplastic glutamine phosphoribosylpyrophosphate amidotransferase, herbicide, X-ray crystallography, competitive inhibition, *Arabidopsis thaliana*, DAS734

In the original article, the authors neglected to include the funder Beijing Natural Science Foundation (5204038) to YiZ.

The authors apologize for this error and state that this does not change the scientific conclusions of the article in any way. The original article has been updated.

## Publisher's Note

All claims expressed in this article are solely those of the authors and do not necessarily represent those of their affiliated organizations, or those of the publisher, the editors and the reviewers. Any product that may be evaluated in this article, or claim that may be made by its manufacturer, is not guaranteed or endorsed by the publisher.

