# Supplementary material for: Crystal Structure of the Chloroplastic Glutamine Phosphoribosylpyrophosphate Amidotransferase GPRAT2 From Arabidopsis thaliana
Source: Front Plant Sci. 2020 Feb 27;11:157. doi: 10.3389/fpls.2020.00157 (PMC7056826; doi:10.3389/fpls.2020.00157)
Supplement: Supplementary file 1 [file DataSheet_1.pdf]

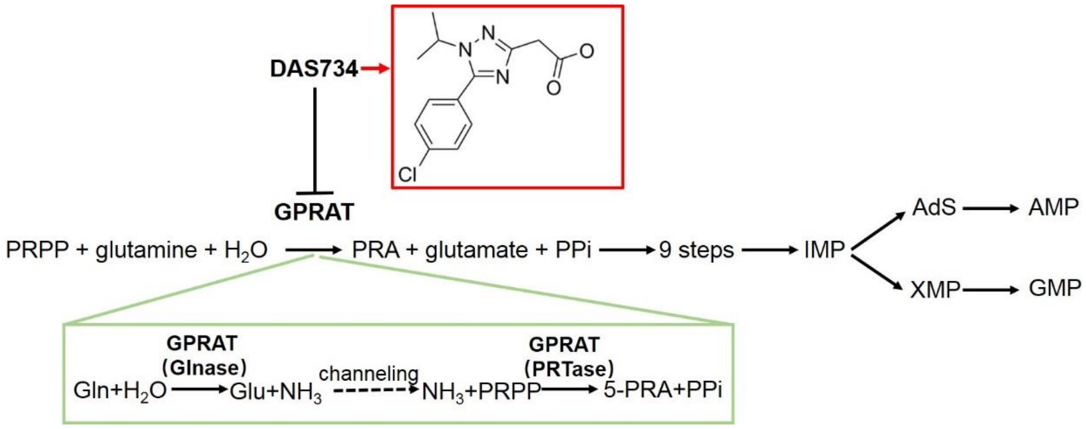

Figure S1 Purine biosynthesis pathway, the reaction catalyzed by GPRAT, and the site of inhibition by DAS734. Abbreviations are defined as follows: PRPP, phosphoribosylpyrophosphate; PRA, phosphoribosylamine; PPi, pyrophosphate; AdS, adenylosuccinate. Summary of reactions catalyzed by GPRAT is indicated in the green box. The structure of DAS734 is highlighted with a red box.

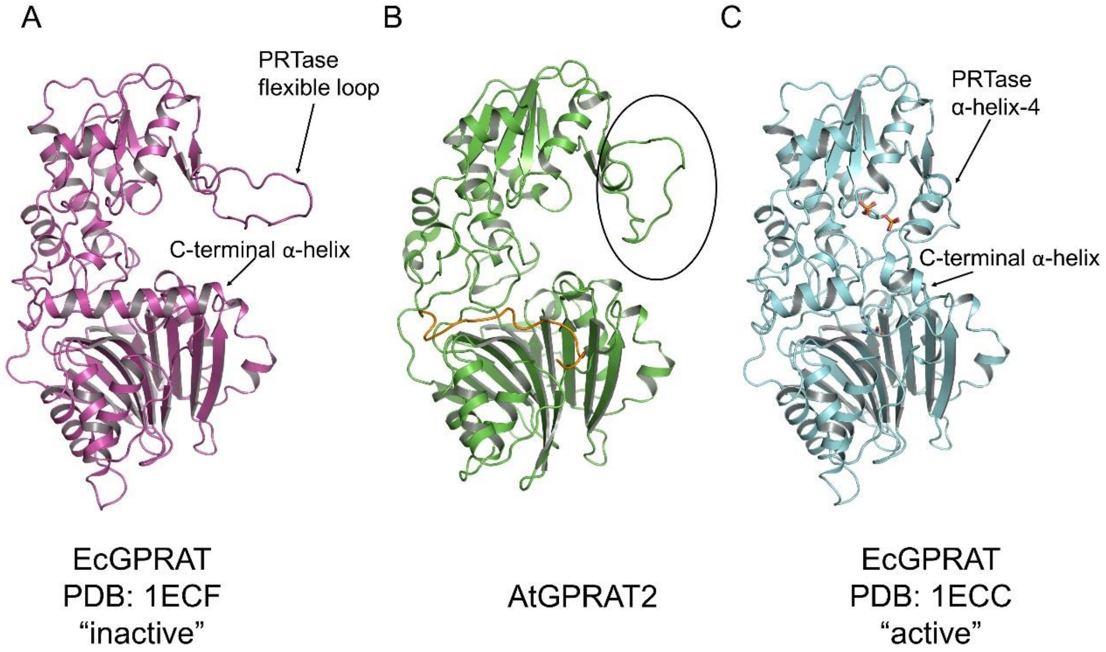

Figure S2 Structural comparison indicates that AtGPRAT2 is in an inactive conformation.

A-C. Cartoon models of AtGPRAT2 and EcGPRAT in active and inactive conformations. The C-terminal helix of EcGPRAT is shown, while the corresponding region in AtGPRAT2 forms a loop and is colored in orange. The PRTase flexible loop region in AtGPRAT2 is marked with a circle. The cPRPP molecule is shown as sticks.

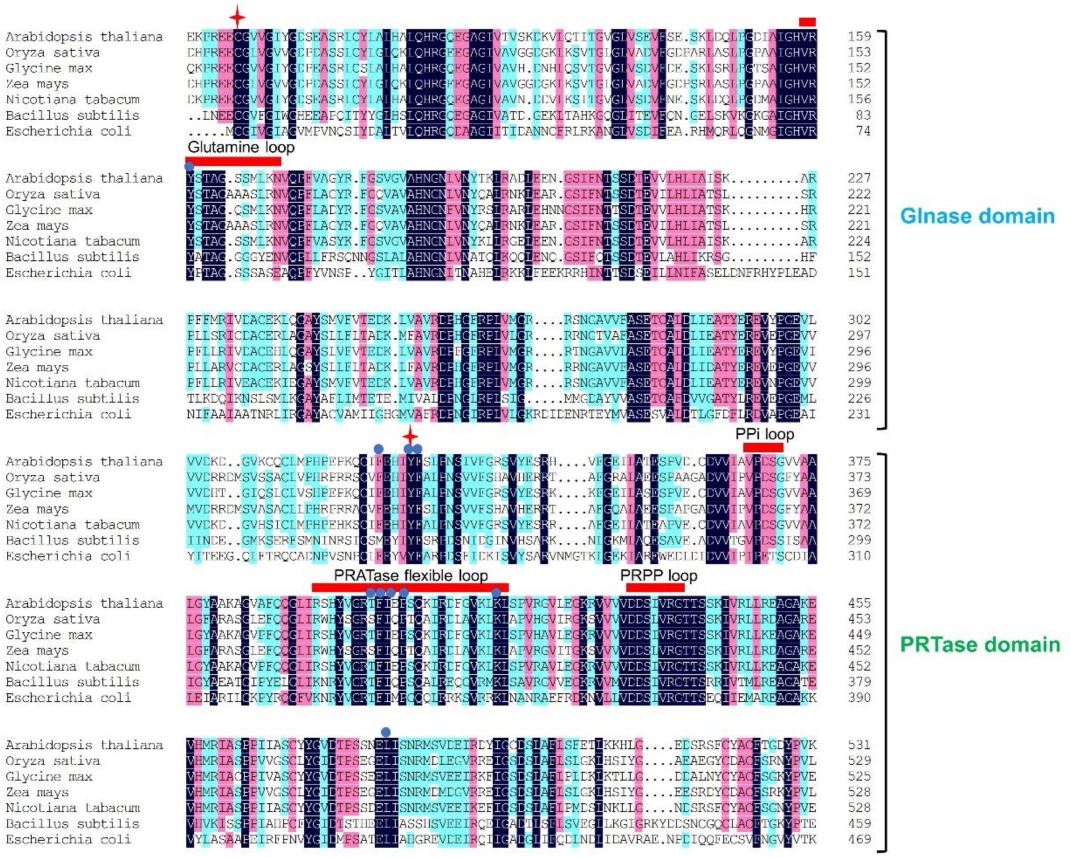

Figure S3 Sequence alignment among AtGPRAT2 and its homologs.

Residues with 100 % identity, over 75 % identity, and over 50 % identity are shaded in dark blue, pink and light blue, respectively. NCBI sequence IDs are: NP_195200.1 (*Arabidopsis thaliana*), XP_015640464.1 (*Oryza sativa*), NP_001238270.2 (*Glycine max*), XP_016440616.1 (*Nicotiana tabacum*), WP_003233947.1 (*Bacillus subtilis*) and WP_113449466.1 (*Escherichia coli*). The N-terminal catalytic Cys and Y329 in AtGPRAT2 are marked with stars and the four loop regions are highlighted. The residues lining the ammonia channel in EcGPRAT are marked with blue dots.

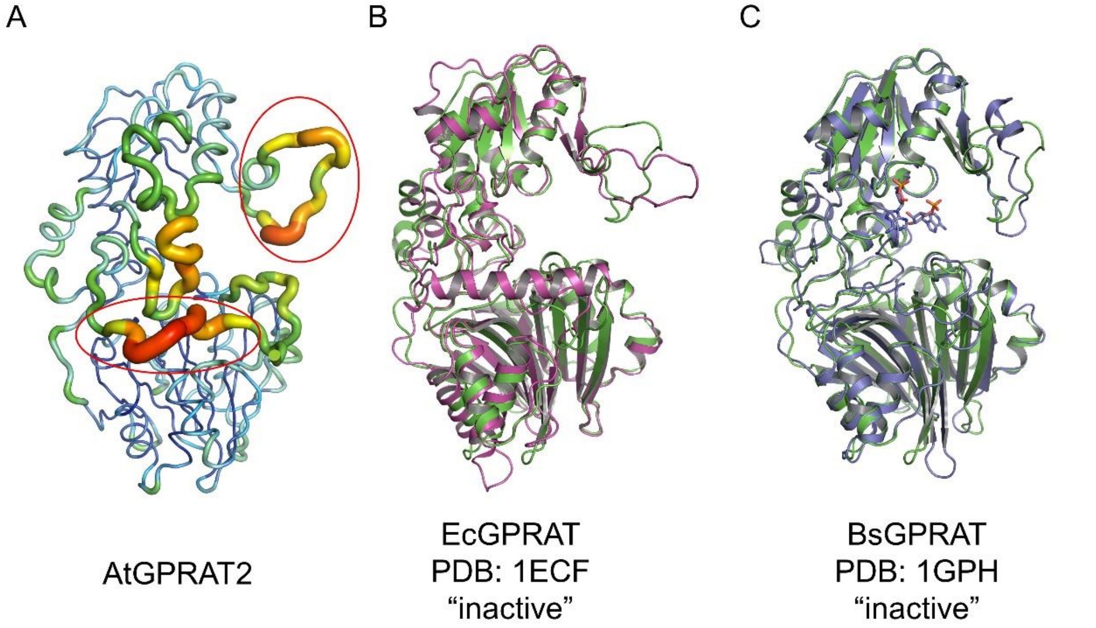

Figure S4 Structural comparison between AtGPRAT2 and bacterial GPRATases. A. Analysis of intramolecular B-factor values in the AtGPRAT2 structure. AtGPART2 is shown in cartoon, with blue representing the lowest and red the highest B-factor values. The size of the tube also reflects the B-factor values: the higher the B-factor, the thicker the tube. The PRATase and C-terminal flexible loop regions are marked in circles. B-C. Structural superimposition of AtGPRAT2 (green), EcGPRAT (magenta) and BsGPRAT (slate). The AMP molecules in the structure of BsGPRAT are shown as sticks.

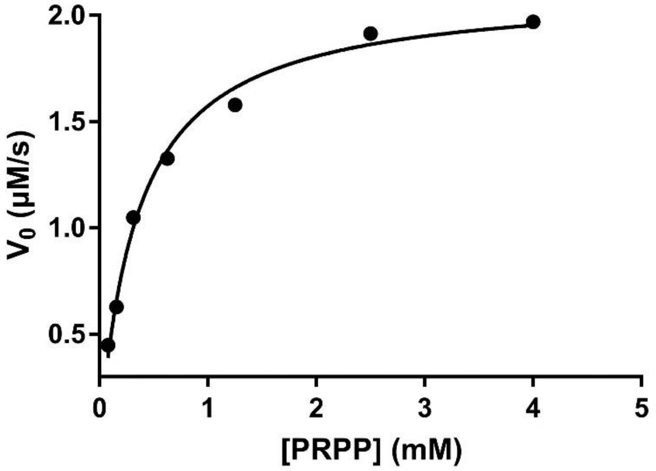

Figure S5 Kinetic analysis of AtGPRAT2

The kinetics data were fitted using GraphPad Prism software and the calculated K_m for PRPP is 0.35 mM.

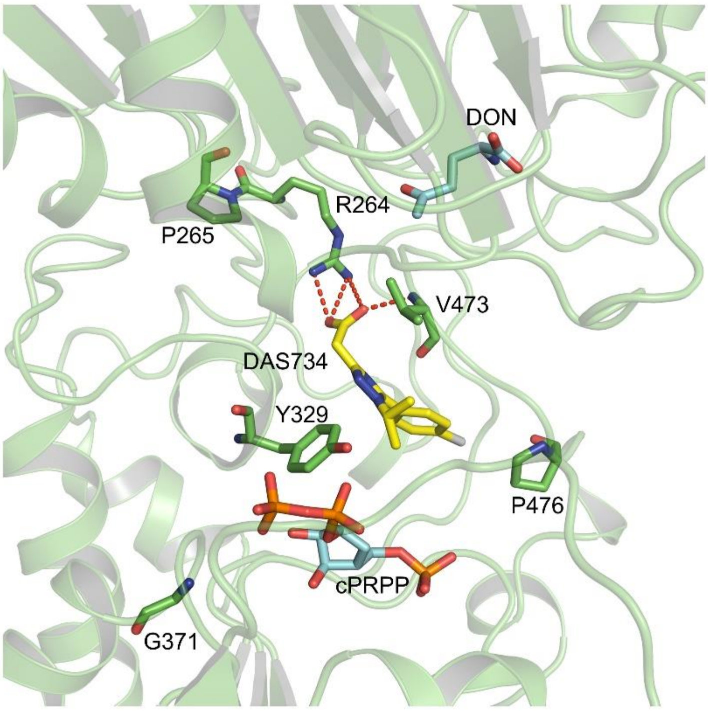

Figure S6 Analysis of the mutation sites showing DAS734 resistance

Structural superimposition of AtGPRAT2 with docked DAS734 and EcGPRAT in the active (PDB: 1ECC) conformation. DAS734, cPRPP and DON are shown as sticks. The

reported mutation residues R264, P265, G371 and P476, the important DAS734 binding residues Y329 and V473 are shown in sticks. Hydrogen bonds are represented by red dashed lines.

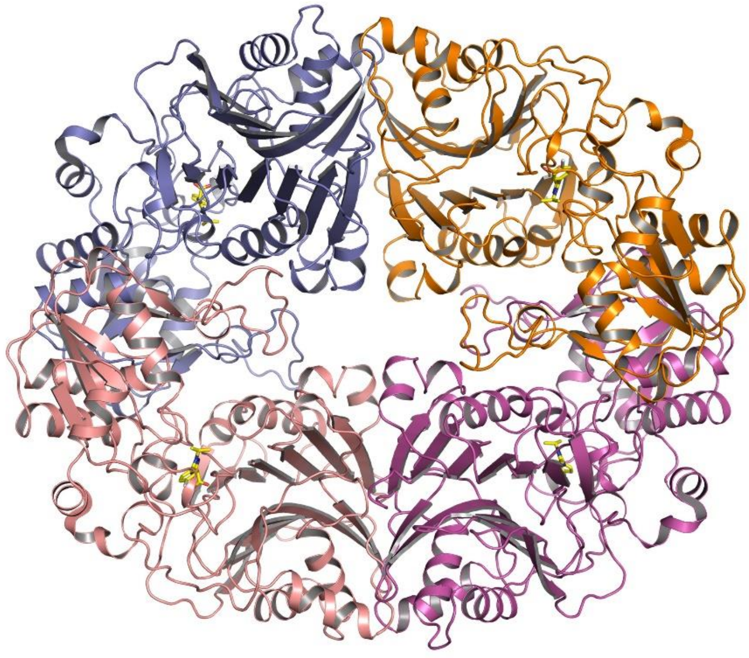

Figure S7 DAS734 does not interfere with the tetramer interface

The GPRAT2 tetramer is shown in cartoon and the docked molecules of DAS734 are shown in sticks.
