# Supplementary material for: Crystal Structure of the Chloroplastic Glutamine Phosphoribosylpyrophosphate Amidotransferase GPRAT2 From Arabidopsis thaliana
Source: Front Plant Sci. 2020 Feb 27;11:157. doi: 10.3389/fpls.2020.00157 (PMC7056826; doi:10.3389/fpls.2020.00157)
Supplement: Supplementary file 2 [file Table_1.docx]

**Table S1 Data collection and refinement statistics of AtGPRAT2**

|  |  | **AtGPRAT2** |
| --- | --- | --- |
| **Data collection** |  |  |
| Space Group |  | P3_1_21 |
| Unit Cell (Å)  α, β, γ (°) |  | 179.74, 179.74, 109.20  90, 90, 120 |
| Wavelength (Å) |  | 0.979 |
| Resolution (Å) |  | 3.07 (3.18-3.07) |
| R_merge_ % |  | 9.9 (73.2) |
| I/sigma |  | 18.3 (3.11) |
| Completeness (%) |  | 99.9 (100.0) |
| Redundancy |  | 6.0 (6.0) |
| **Refinement** |  |  |
| R_work_ |  | 0.1933 |
| R_free_ |  | 0.2131 |
| No. protein atoms |  | 6887 |
| Overall B factors: |  | 58.56 |
| RMSD bond lengths |  | 0.007 |
| RMSD bond angles |  | 0.91 |
| Ramachandran plot statistics (%) |  |  |
| In favored regions |  | 95.16 |
| In allowed regions |  | 4.62 |
| Outliers |  | 0.23 |
| PDB code |  | 6LBP |

Values in parentheses are for the highest resolution shell. *R_merge_*=Σ_h_Σ_i_|*I_h,i_*-*I_h_*|/Σ_h_Σ_i_*I_h,i_*, where *I_h_* is the mean intensity of the *i* observations of symmetry related reflections of *h*. *R*=Σ|*F_obs_*-*F_calc_*|/Σ*F_obs_*, where *F_calc_* is the calculated protein structure factor from the atomic model (R_free_ was calculated with 5% of the reflections selected).
